# Supplementary material for: CBP/β-Catenin/FOXM1 Is a Novel Therapeutic Target in Triple Negative Breast Cancer
Source: Cancers (Basel). 2018 Dec 19;10(12):525. doi: 10.3390/cancers10120525 (PMC6315782; doi:10.3390/cancers10120525)
Supplement: Supplementary file 1 [file cancers-10-00525-s001.pdf]

Supplementary Materials: Cbp/B-Catenin/Foxm1 Is A Novel Therapeutic Target in Triple Negative Breast Cancer

Alexander Ring, Cu Nguyen, Goar Smbatyan, Debu Tripathy, Min Yu, Michael Press, Michael Kahn and Julie E. Lang

Supplementary figures

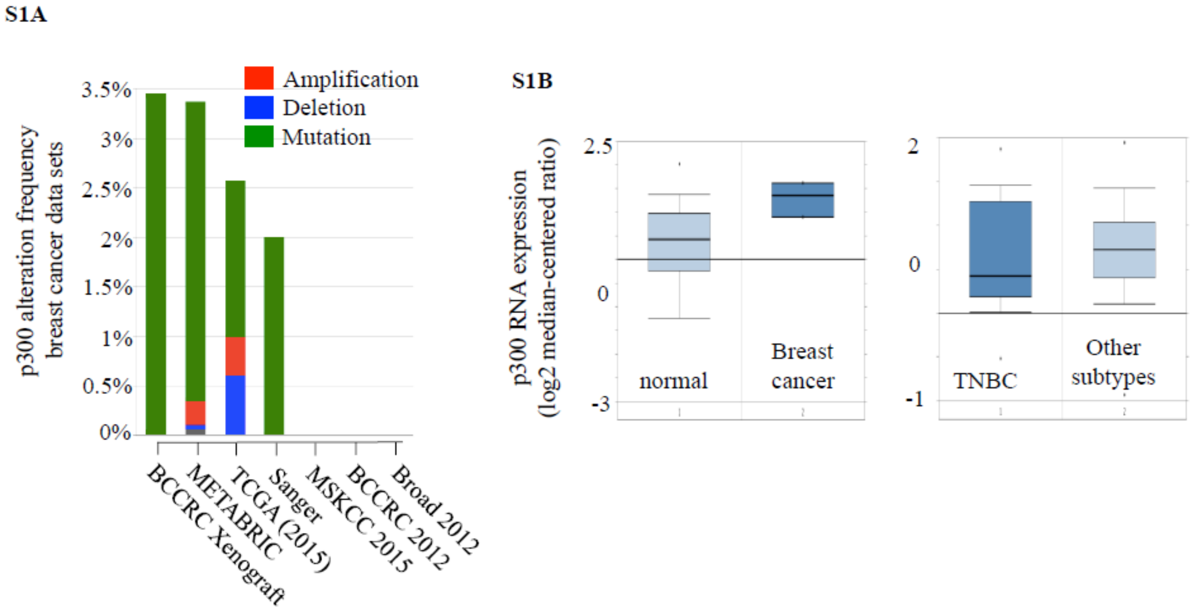

Figure S1. p300 genomic and transcriptional alterations in BC. (**A**)Ten publicly available data sets show genetic alterations in p300 in breast cancer (cBioPortal). (**B**) RNA expression levels of p300 in the TCGA BC data set (n = 593); left box plot: normal breast tissue compared to BC (2.1-fold BC vs. normal, p = 0.008), right box plot TNBC compared to BC other subtypes (-1.1-fold TNBC vs. others, p = 0.839) (Oncomine database).

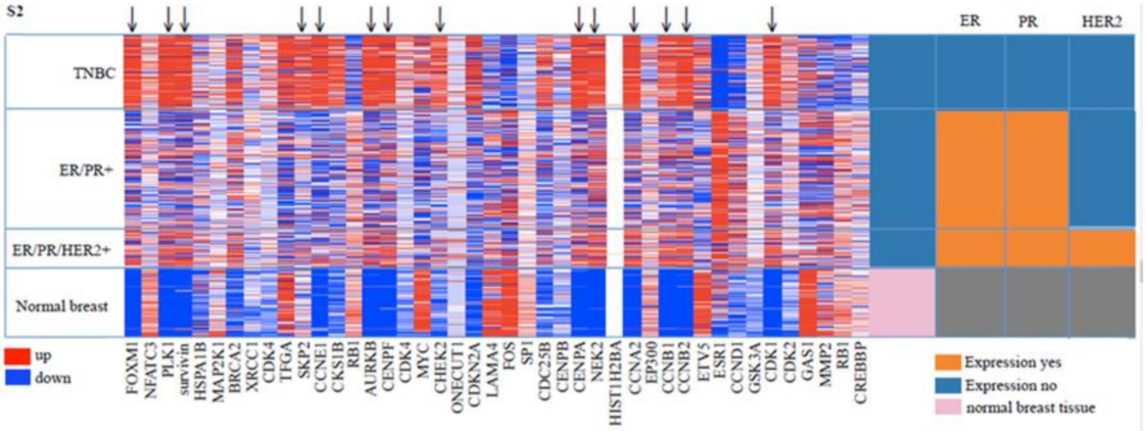

Figure S2. TCGA data analysis shows that FOXM1 and its target genes are over-expressed in TNBC. TCGA breast cancer data dataset visualization via the Santa Cruz Cancer Genome Browser (individual BC cases represented in rows, genes represented in columns) showing a FOXM1 transcription network in different breast cancer subtypes and normal breast tissue (n = 817 samples, 82 TNBC). The arrows indicate highly expressed genes in TNBC samples in the TCGA BC data set

that are down-regulated in MDA-MB-231 cells treated with ICG-001 compared to DMSO control (shown in Figure 4A).

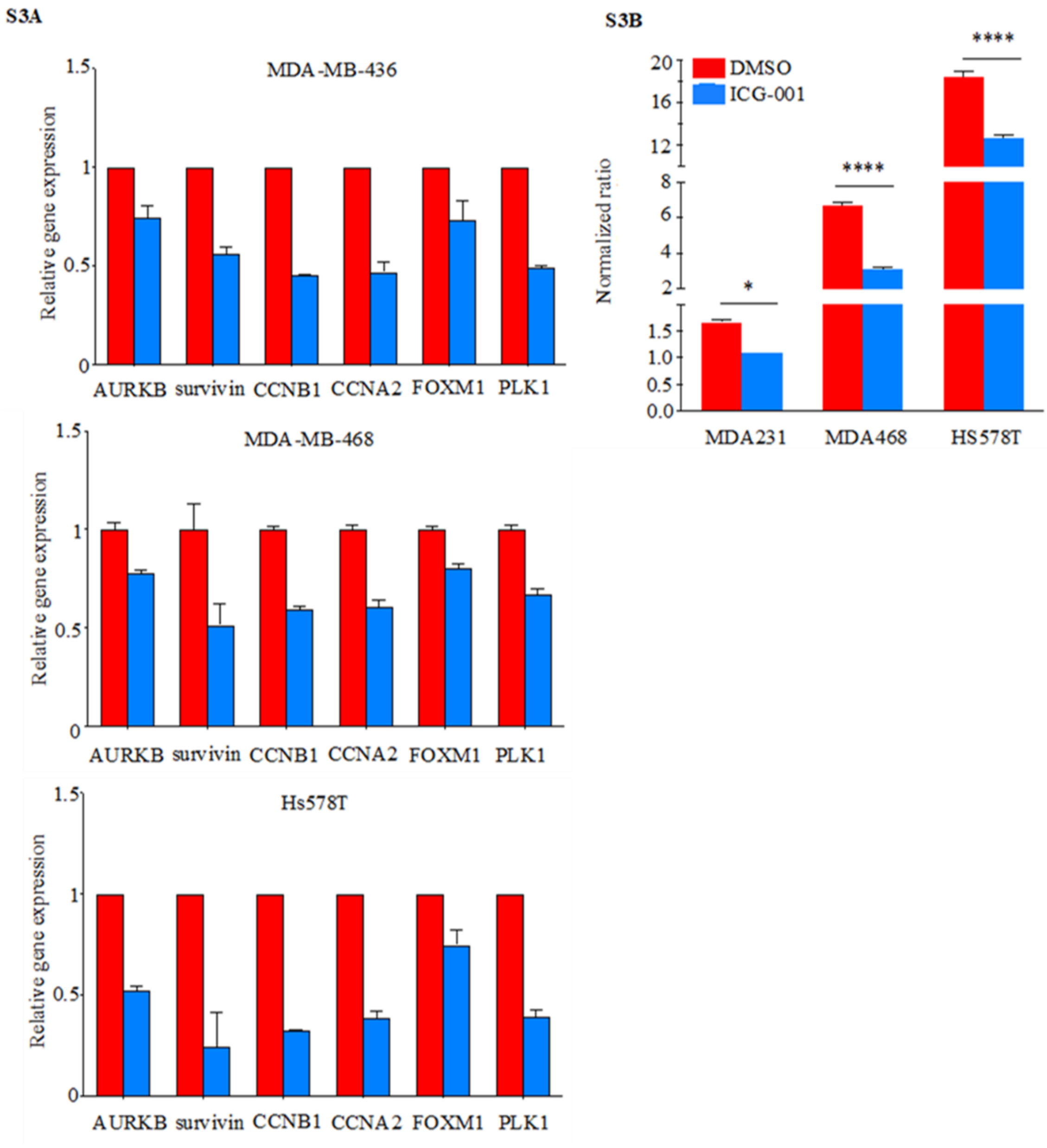

Figure S3. FOXM1 reporter activity and target gene expression in three additional TNBC cell lines. (**A**) FOXM1 target gene expression in three TNBC cell lines treated for 24 h with 10 µM ICG-001 or DMSO vehicle control. (**B**) FOXM1luc$^{Firefly/Renilla}$ reporter for three TNBC cell lines (MDA-MB-231, MDA-MB-468, Hs578T) treated for 24 h with either 10 µM ICG-001 or DMSO vehicle control (bars represent normalized ratios of FOXM1 Firefly luciferase to control vector Renilla luciferase expression; *N* = 3 per condition per cell line) (* $p < 0.05$, **** $p < 0.0001$).

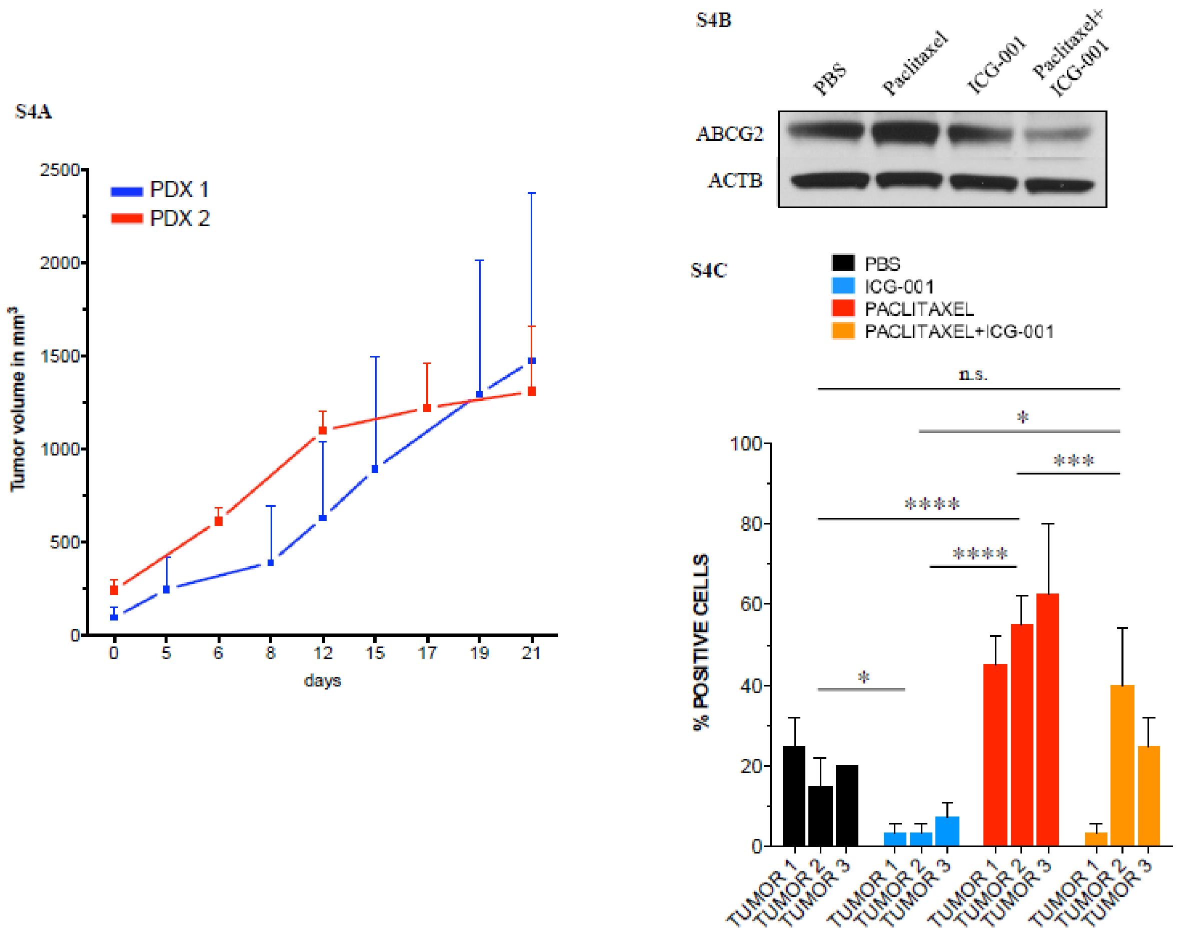

Figure S4. Protein quantification of FOXM1 and ABCG2 in PDX2 bearing mice post treatment. (**A**) Growth curves for PBS control treated xenograft tumors. (**B**) Western blot protein quantification post treatment (primary xenograft) for ABCG2 in tumors from mice bearing PDX2. (**C**) Immunohistochemistry post treatment (primary xenograft) for FOXM1 on tumors from mice bearing PDX1 (3 Tumors per condition, 2 sections per tumor) (* $p < 0.05$, *** $p < 0.001$, **** $p < 0.0001$, Statistically insignificant results are listed n.s.).

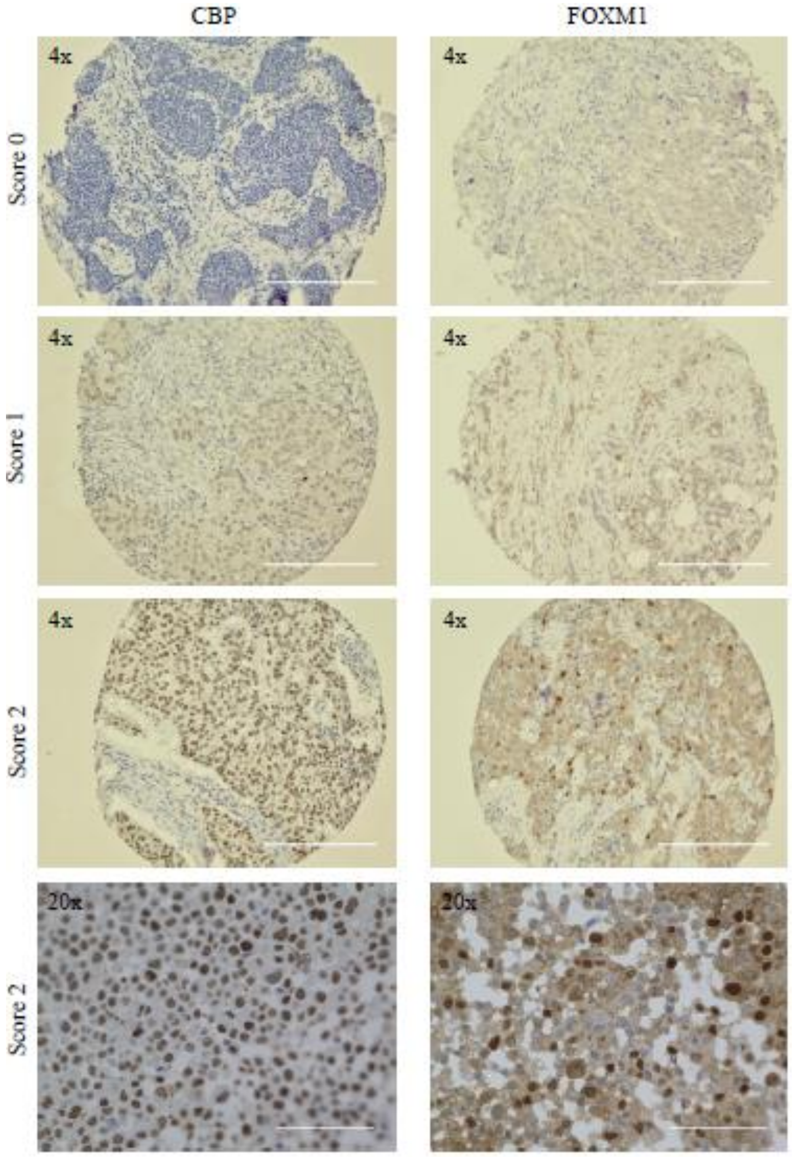

Figure S5. Representative staining of TMA slides for FOXM1 and CBP; top three panels–4× magnification, scale bar = 200 µm, bottom panel–20× magnification (scale bar = 100 µm).

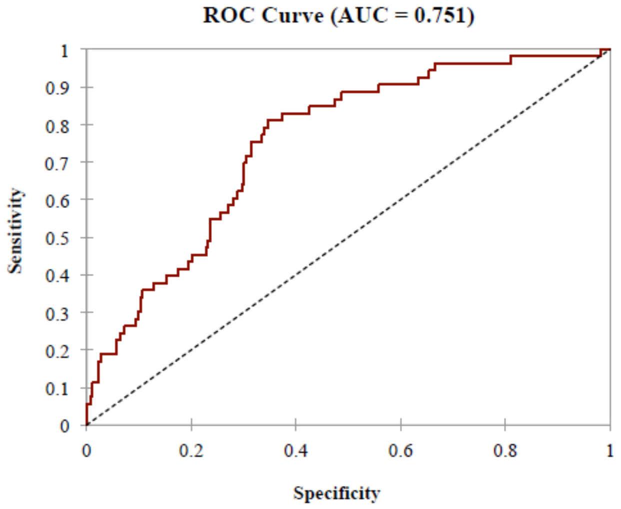

Figure S6. Receiver operating characteristic (ROC) curve for FOXM1 as a biomarker for TNBC and high grade (III) tumors.

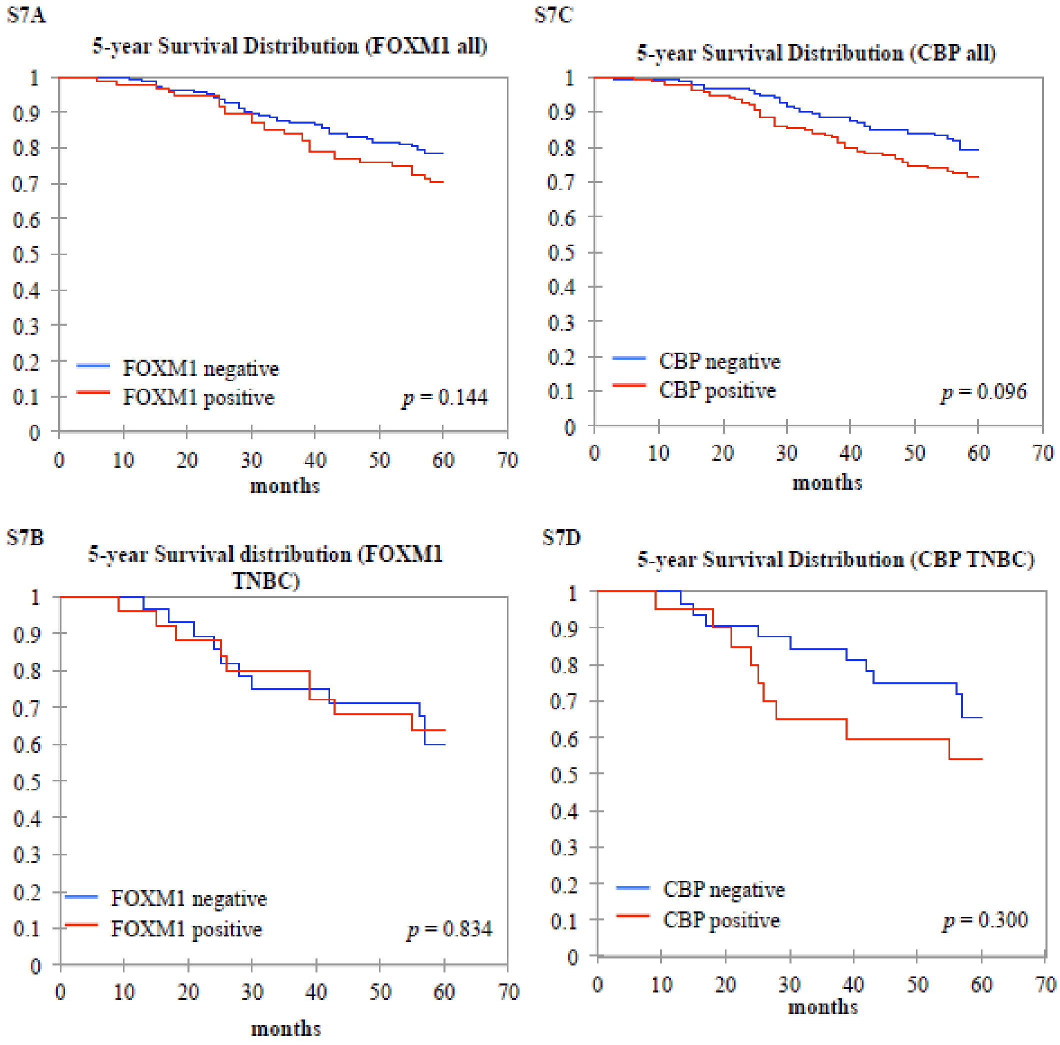

Figure S7. TMA Kaplan Meier survival analysis in BC using FOXM1 and CBP. (**A**) OS survival based on FOXM1 expression in TMA all BC patients (n = 316). (**B**) OS survival based on FOXM1 expression in TMA TNBC patients (n = 52). (**C**) OS survival based on CBP expression in TMA all BC patients (n = 316). (**D**) OS survival based on CBP expression in TMA TNBC patients (n = 52).

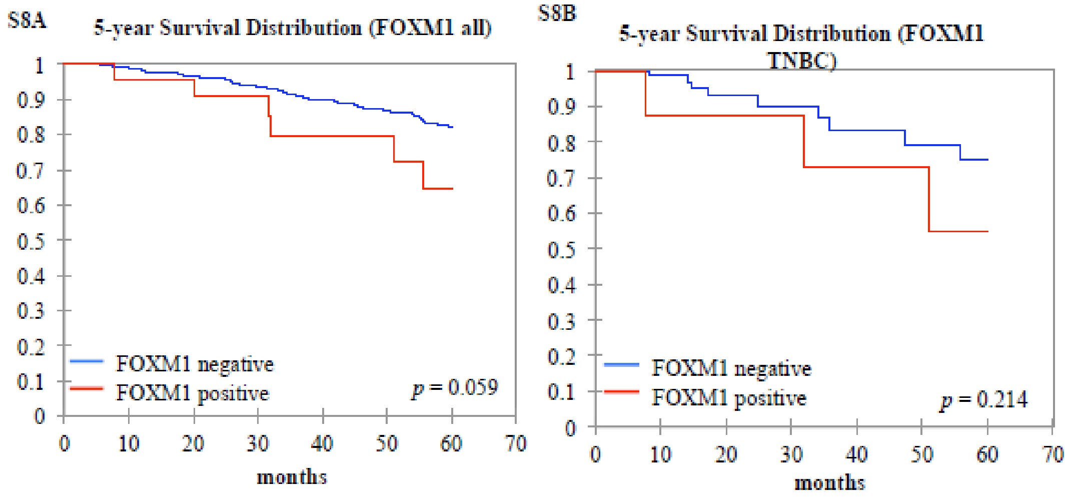

Figure S8. TCGA Kaplan Meier survival analysis in BC using FOXM1 and CBP. (**A**) OS survival based on FOXM1 expression in TCGA BC patients of all subtypes (n = 817). (**B**) OS survival based on FOXM1 expression in TCGA TNBC patients (n = 82).

Supplementary Tables

Table S1. Differential FOXM1-driven gene expression in MDA-MB-231 after 48 h siRNA mediated gene knockdown.

Gene/KD Condition	FOXM1+ CTNNB1	FOXM1+CBP	CBP+ CTNNB1	FOXM1+CBP+ CTNNB1
CBP	n.s.	$p < 0.001$	$p < 0.01$	$p < 0.01$
CTNNB1	$p < 0.001$	n.s.	$p < 0.001$	$p < 0.001$
FOXM1	$p < 0.001$	$p < 0.001$	n.s.	$p < 0.001$
AURKB	$p < 0.05$	n.s.	n.s.	n.s.
CCNB1	$p < 0.001$	n.s.	$p < 0.05$	n.s.
CCNA2	$p < 0.001$	$p < 0.001$	$p < 0.05$	$p < 0.001$
BIRC5	$p < 0.001$	$p < 0.05$	n.s.	$p < 0.05$
PLK1	$p < 0.05$	n.s.	n.s.	n.s.

Table S2. Mammosphere size difference after 48 h siRNA gene knockdown.

Cell Lines	Sphere Size	# of Spheres DMSO	# of Spheres ICG-001
MDA-MB-436	Small	66 ± 4	24 ± 6
	Large	26 ± 3	0
CAL51	Small	33 ± 3	9 ± 1
	Large	8 ± 2	0
Hs578T	Small	28 ± 3	17 ± 3
	Large	23 ± 6	3 ± 1
SUM149	Small	32 ± 3	20 ± 2
	Large	27 ± 2	0

Table S3. Comparison of tumor volume post 24 days; primary implantation MDA-MB-468 xenograft ($N = 4$ mice per group).

Treatment Comparison	Mean difference (mm^3)	Significance
PBS vs. ICG-001	55.82	n.s.
PBS vs. paclitaxel + PBS	548.1	$p < 0.01$
PBS vs. paclitaxel + ICG-001	1097	$p < 0.0001$
ICG-001 vs. paclitaxel + PBS	492.2	$p < 0.01$
ICG-001 vs. paclitaxel + ICG-001	1041	$p < 0.0001$
Paclitaxel + PBS vs. paclitaxel + ICG-001	549.1	$p < 0.01$

Table S4. Comparison of tumor volume post 24 days; secondary implantation MDA-MB-468 xenograft ($n = 5$ mice per treatment condition).

Treatment Comparison	Mean Difference (mm^3)	Significance
PBS vs. ICG-001	−354.6	n.s.
PBS vs. paclitaxel + PBS	−683.4	$p < 0.05$
PBS vs. paclitaxel + ICG-001	753.5	$p < 0.05$
ICG-001 vs. paclitaxel + PBS	−328.9	n.s.
ICG-001 vs. paclitaxel + ICG-001	1108	$p < 0.001$
Paclitaxel + PBS vs. paclitaxel + ICG-001	1437	$p < 0.0001$

Table S5. Clinical characteristic for patient 1 and 2 used for PDX models of TNBC in NGS mice (NeoChemo–neaoadjuvant chemotherapy, AC–Doxorubicin and Cyclophosphamide, T–Taxol (paclitaxel), MRM–modified radical mastectomy).

<table>
<tr><th>Patient</th><th>Histology</th><th>Stage</th><th>Grade</th><th>TNM Stage</th><th>Treatment</th><th>Relapse</th><th>Survival</th></tr>
<tr><td>1</td><td rowspan="2">Infiltrating Ductal Carcinoma</td><td rowspan="2">IIb</td><td rowspan="2">poorly differentiated</td><td rowspan="2">yT3N0Mx</td><td rowspan="2">NeoChemo AC->T Surgery: MRM Radiation</td><td>No</td><td>Alive</td></tr>
<tr><td>2</td><td>Yes</td><td>Deceased</td></tr>
</table>

Table S6. Comparison of tumor volume post 24 days; primary implantation patient 1 PDX ($n = 4$ mice for ICG-001, $n = 5$ per each other treatment conditions).

Treatment Comparison	Mean Difference (mm³)	Significance
PBS vs. ICG-001	112.1	n.s.
PBS vs. paclitaxel + PBS	1206	$p < 0.01$
PBS vs. paclitaxel + ICG-001	1332	$p < 0.01$
ICG-001 vs. paclitaxel + PBS	1094	$p < 0.05$
ICG-001 vs. paclitaxel + ICG-001	1220	$p < 0.05$
Paclitaxel + PBS vs. paclitaxel + ICG-001	126.1	n.s.

Table S7. Comparison of tumor volume post 24 days; secondary implantation patient 1 PDX ($n = 3$ mice for ICG-001, $n = 4$ per each other treatment condition).

Treatment Comparison	Mean Difference (mm³)	Significance
PBS vs. ICG-001	753.8	$p < 0.01$
PBS vs. paclitaxel + PBS	656.2	$p < 0.01$
PBS vs. paclitaxel + ICG-001	516	$p < 0.05$
ICG-001 vs. paclitaxel + PBS	−97.64	n.s.
ICG-001 vs. paclitaxel + ICG001	−237.8	n.s.
Paclitaxel + PBS vs. paclitaxel + ICG-001	−140.2	n.s.

Table S8. Comparison of tumor volume post 24 days; primary implantation patient 2 PDX ($n = 4$ mice each for PBS and ICG-001, $n = 6$ mice each for Paclitaxel + PBS and Paclitaxel + ICG-001).

Treatment Comparison	Mean Difference (mm³)	Significance
PBS vs. ICG-001	655.8	$p < 0.05$
PBS vs. paclitaxel + PBS	155.1	n.s.
PBS vs. paclitaxel + ICG-001	642.4	$p < 0.05$
ICG-001 vs. paclitaxel + PBS	810.9	$p < 0.01$
ICG-001 vs. paclitaxel + ICG-001	1298	$p < 0.0001$
Paclitaxel + PBS vs. paclitaxel + ICG-001	487.3	n.s.

Table S9. Comparison of tumor volume post 24 days; secondary implantation patient 2 PDX ($n = 5$ mice per group).

Treatment Comparison	Mean difference (mm³)	Significance
PBS vs. ICG-001	−74.42	n.s.
PBS vs. paclitaxel + PBS	274.7	n.s.
PBS vs. paclitaxel + ICG-001	554.1	$p < 0.001$
ICG-001 vs. paclitaxel + ICG-001	628.5	$p < 0.0001$
ICG-001 vs. paclitaxel + PBS	349.1	$p < 0.05$
Paclitaxel + PBS vs. paclitaxel + ICG-001	279.5	n.s.

Table S10. Statistical analysis (Chi square statistics–FOXM1 as dependent variable).

Variable	FOXM1 (+)	FOXM1 (−)	Total	% Positive	*p*-Value
Differentiation					
Grade 1	7	39	46	15	
Grade 2	41	110	151	27	
Grade 3	47	79	126	37	**0.013**
Subtype					
TNBC	25	28	53	47	**<0.001**
ER+	4	20	24	17	0.177
ER/PR+	43	134	177	24	0.045
HER2+	13	30	43	30	0.8

Table S11. Chi square statistics (CBP as dependent variable).

Variable	CBP (+)	CBP (−)	Total	% Positive	*p*-Value
Subtype					
TNBC	20	33	53	38	0.329
ER+	12	12	24	50	0.521
ER/PR+	72	105	177	41	0.192
HER2+	26	17	43	60	**0.017**
Nodal status					
N0	40	76	116	35	
N1	96	110	206	47	**0.034**

Table S12. TMA BC subtype distribution.

Subtype	Total Number	%
ER+/PR-	24	7.7
ER-/PR+	11	3.5
ER/PR+	176	56.2
HER2+	49	15.7
TNBC	53	16.9

Table S13. 316 tumor cores evaluated for FOXM1 and CBP staining. Staining scoring 0 = negative, 1 = weakly positive, 2 = strongly positive (nuclear staining).

Protein	FOXM1		CBP	
	Total Number	%	Total Number	%
Positive (2)	95	30.4	136	44.7
Weakly positive (1) or Negative	218	69.6	168	55.3

Table S14. Multivariate Logistic Regression (FOXM1 dependent variable).

Source	*p*-Value	Odds Ratio	Odds Ratio Lower Bound (95%)	Odds Ratio Upper Bound (95%)
TNBC	**0.034**	2.02	1.06	3.85
Differentiation				
Grade II	**0.023**	2.79	1.15	6.75
Grade III	**0.01**	3.3	1.34	8.12
Radiation therapy	**0.04**	1.74	1.03	2.96

Table S15. Cox Regression analysis (calculated using vital status plus overall survival in months).

Variable	*p*-Value	Hazard Ratio	Odds Ratio Lower Bound (95%)	Odds Ratio Upper Bound (95%)
TNBC	**0.023**	1.63	1.07	2.49
Age ≤ 50 years	**<0.0001**	0.035	0.23	0.53
Differentiation				
Grade III	0.051	1.63	1	2.65
Tumor stage				
T2	0.053	1.43	1	2.04

Table S16. TMA data set all cases mean OS based on FOXM1 expression.

Marker Expression	Mean Survival Time (OS)	Standard Deviation	Lower Bound (95%)	Upper Bound (95%)
FOXM1 (+)	50.947	1.389	48.226	53.669
FOXM1 (−)	51.962	0.788	50.418	53.505

Statistic	Observed Value	Critical Value	*p*-Value	Alpha
Log-rank	2.132	3.841	0.144	0.050

Table S17. TMA data set TNBC cases mean OS based on FOXM1 expression.

Marker Expression	Mean Survival Time (OS)	Standard Deviation	Lower Bound (95%)	Upper Bound (95%)
FOXM1 (+)	45.960	3.129	39.827	52.093
FOXM1 (−)	47.793	2.994	41.924	53.661

Statistic	Observed Value	Critical Value	*p*-Value	Alpha
Log-rank	0.044	3.841	0.834	0.050

Table S18. TMA data set all cases mean OS based on CBP expression.

Marker Expression	Mean Survival Time (OS)	Standard Deviation	Lower Bound (95%)	Upper Bound (95%)
CBP (+)	50.708	1.175	48.405	53.011
CBP (−)	52.657	0.837	51.016	54.298

Statistic	Observed Value	Critical Value	*p*-Value	Alpha
Log-rank	2.772	3.841	0.096	0.050

Table S19. TMA data set TNBC cases mean OS based on CBP expression.

Marker Expression	Mean Survival Time (OS)	Standard Deviation	Lower Bound (95%)	Upper Bound (95%)
CBP (+)	42.433	3.829	34.929	49.937
CBP (−)	49.719	2.560	44.701	54.737

Statistic	Observed Value	Critical Value	*p*-Value	Alpha
Log-rank	1.075	3.841	0.300	0.050

Table S20. TCGA RPPA data set all cases mean OS based on FOXM1 expression.

Marker Expression	Mean Survival Time (OS)	Standard Deviation	Lower Bound (95%)	Upper Bound (95%)
FOXM1 (+)	48.878	3.186	42.635	55.122
FOXM1 (−)	55.302	0.508	54.306	56.299

Statistic	Observed Value	Critical Value	*p*-Value	Alpha
Log-rank	3.577	3.841	0.059	0.050

Table S21. TCGA RPPA data set TNBC cases mean OS based on FOXM1 expression.

Marker Expression	Mean Survival Time (OS)	Standard Deviation	Lower Bound (95%)	Upper Bound (95%)
FOXM1 (+)	42.889	6.545	30.061	55.718
FOXM1 (−)	50.074	1.955	46.243	53.905

Statistic	Observed Value	Critical Value	*p*-Value	Alpha
Log-rank	1.543	3.841	0.214	0.050

Table S22. Cell lines and culture media.

Cell Line	Culture Medium
MDA-MB-231<br>Hs578T<br>MDA-MB-436<br>SUM149	Dulbecco's Modified Eagle Medium (DMEM) (Thermo Fisher Scientific, Canoga Park, CA) + 10% FBS (Atlanta Biologicals, Flowery Branch, GA) + 1% Antibiotic-Antimycotic mix (Thermo Fisher Scientific)
MDA-MB-468	1:1 mixture of DMEM and Ham's F12 + 10% FBS + 1% Antibiotic-Antimycotic
CAL51	DMEM + 20% FBS + 1% Antibiotic-Antimycotic

Table S23. Genes and primer sequences for qPCR.

NCBI Gene Symbol	Forward Primer (5′→3′)	Reverse Primer (5′→3′)
FOXM1	CGTCGGCCACTGATTCTCAAA	GGCAGGGGATCTCTTAGGTTC
CREBBP (CBP)	CAACCCCAAAAGAGCCAAACT	CCTCGTAGAAGCTCCGACAGT
β-catenin	CATCTACACAGTTTGATGCTGCT	GCAGTTTTGTCAGTTCAGGGA
AURKB	CAGTGGGACACCCGACATC	GTACACGTTTCCAAACTTGCC
ABCG2	ACGAACGGATTAACAGGGTCA	CTCCAGACACACCACGGAT
Survivin	AGCCCTTTCAAGGACCAC	GCACTTTCTTCGCAGTTTCC
CCNA1	ACATGGATGAACTAGAGCAGGG	GAGTGTGCCGGTGTCTACTT
CCNB2	CCGACGGTGTCCAGTGATTT	TGTTGTTTTGGTGGGTTGAACT
CD24	CTCCTACCCACGCAGATTTATTC	AGAGTGAGACCACGAAGAGAC
CD44	CTGCCGCTTTGCAGGTGTA	CATTGTGGGCAAGGTGCTATT
MDR1	TTGCTGCTTACATTCAGGTTTCA	AGCCTATCTCCTGTCGCATTA
PLK1	AAAGAGATCCCGGAGGTCCTA	GGCTGCGGTGAATGGATATTTC
GAPDH	GGTGCTGAGTATGTCTGTGA	ACAGTCTTCTGGGTGGCAGT
PPIA	CCTAAAGCATACGGGTCCTG	TTTCACTTTGCCAAACACCA

Table S24. Clinical variables used for association studies with FOXM1 and CBP protein levels in the TMA analysis.

Variable	Values	Type
Marker (FOXM1, CBP)	1 = positive<br>0 = negative	Categorical
Subtype	TNBC<br>ER/PR+<br>ER+<br>HER2+	
Age	≤ 50 years<br>> 50 years	
Stage	T1<br>T2<br>T3	
Grade	Grade I<br>Grade II<br>Grade III	
N<br>(lymph node metastasis)	N0 = no lymph node metastasis<br>N1 = yes lymph node metastasis	
Chemotherapy	Yes<br>No	
Radiation therapy	Yes<br>No	
Vital status	Alive<br>Dead	
Tumor size	cm	Numerical
Overall survival	Days	

© 2018 by the authors. Licensee MDPI, Basel, Switzerland. This article is an open access article distributed under the terms and conditions of the Creative Commons Attribution (CC BY) license (http://creativecommons.org/licenses/by/4.0/).
